# Cell and gene therapy regulatory, pricing, and reimbursement framework: With a focus on South Korea and the EU

**DOI:** 10.3389/fpubh.2023.1109873

**Published:** 2023-02-24

**Authors:** SungKyung Lee, Jong Hyuk Lee

**Affiliations:** ^1^Center for Growth Engine Industries, Korea Institute for Industrial Economics and Trade (KIET), Sejong, Republic of Korea; ^2^College of Pharmacy, Chung-Ang University, Seoul, Republic of Korea

**Keywords:** cell and gene therapy, regulation, reimbursement, industrial policy, pricing, regulatory harmonization, innovative medicine, patient accessibility

## Abstract

Ever since relevant bioengineering technologies have sufficiently matured to the platformizable commercialization stage, a slew of money has flocked to the cell and gene therapy market over the last few years, resulting in an abundance of clinical studies in the field. Newer modalities have brought up a string of regulatory and legislative tasks, such as developing guidelines and legislative rules to systematically regulate newer pharmaceutical products. Accordingly, another layer of legislation and guidelines tailored for cell and gene therapies has been introduced and is expected to evolve on par with technological progress. Furthermore, authorities have shifted to pricing and reimbursement policies that can share risks for cost and outcome among stakeholders altogether, such as developers and the government, while expanding the accessibility of patients to innovative cell and gene therapies. This review attempts to capture the salient regulatory features of the cell and gene therapy market in the context of South Korea and the European Union and points out where two sovereign entities currently stand on each policy element and how each tackles regulatory challenges. We can observe the converging trend where regulatory, pricing and reimbursement rules of adjoining countries in the supranational union or member countries of a consortium are getting more aligned. Evidently, concerted efforts to share regulatory science knowledge and embrace reference pricing have played their parts. The authors argue that policy priorities should be placed on initiatives to harmonize with other medical authorities to better the rights of patients and clear out the uncertainties of developers, ultimately to share and advance regulatory science and layout forward-looking policies at opportune times.

## 1. Introduction

With the furtherance of bioengineering technologies, many cell and gene therapies (CGTs) have come to fruition recently, which subsequently has made much of venture capital investment in the biotechnology sector turn their eyes to this aspiring modality. Between 2019 and 2021, 15.3 billion USD out of the total 51.8 billion USD of venture capital biotech funding (~30%) has been earmarked for CGTs (1). Thus far, CGTs have mostly targeted cancers, rare diseases, or severe diseases for which alternative medicines have not been developed yet, but nowadays, their coverage has expanded to other severe diseases in an attempt to ultimately corner a market as safer and more effective alternatives.

Since CGTs target genetic entities, if used appropriately, CGTs can cure genetic causes entirely in one or two shots, but they can also induce genetic distortions and immunological responses along the way. Due to such clinical uncertainties, many governments have mandated to trace every prescribed patient for years and set up a new set of laws and regulations that specifically govern CGTs. As of now, regulations for cell and gene therapies are not quite aligned across countries, which acts like non-tariff barriers to trade and stifles the entrance of innovative medicines.

To ease the hefty financial burden that firms entering the market have to bear, governments have minted a series of industrial policies so as to attain a technological edge in advance in the emerging market. Relatively smaller target populations and research and development (R&D) sunk costs make CGTs pricier. Hence, governments are in search of better pricing and reimbursement (P&R) mechanisms to advance the accessibility of patients to innovative medicines with potential clinical benefits.

As more and more expensive CGTs enter the market, governments have begun to feel the budgetary burden. Especially in South Korea, the number of patients who filed for expensive drugs exceeding ~0.2 million USD (0.3 billion KRW) has tripled from 147 in 2016 to 410 in 2020, with total claims filed quadrupling from 26 million USD (37 billion KRW) to 102 million USD (147 billion KRW) over the same period (2). As of 2021, the proportion of the total claims for expensive drugs over the total reimbursement amount claimed is only 0.5%. However, as societies get more gray, more and more expensive drugs are entering the market at an astonishing growth rate, and handling those budgetary concerns in advance will be an important policy priority.

Consequently, streamlining and harmonizing a set of regulations that can advance access to innovative medicines will nurture the ecosystem of the burgeoning industry naturally, which in return results in Pareto improvement as a whole society. In that regard, this review traces the current regulatory landscape of the cell and gene therapy market especially focused on the comparison between South Korea and the European Union (EU) to derive policy implications. South Korea and the EU bear similarities in much of pricing and reimbursement government frameworks in that authorities have more bargaining power than private market players in pricing and reimbursement realm, unlike in the US. More importantly, the Health Technology Assessment (HTA) system of South Korea operates in a similar way to that of the five European countries (Italy, the UK, France, Germany, and Spain; EU5) and especially the UK. Plus, the P&R decisions of EU5 have a huge influence on the HTA assessment of South Korea since South Korea refers to the prices of the EU5 including the prices of the other two countries, the US and Japan. In addition, because of clinical uncertainties of CGTs, risk-sharing agreements (RSAs) are widely adopted in tandem with CGTs in South Korea and the EU5, which makes policy alignment and harmonization between the two a plausible possibility. Hence, our comparative analysis bears witness to the present and future of the regulatory landscape in those two regions.

The order of content is like the following: First, we provide a brief overview of CGT R&D and market trends in South Korea and the EU. After that, we discuss government regulations and industrial policies in the EU and South Korea, followed by the analysis of respective P&R policies where the primary focus will be placed on the comparison of South Korea and the EU5. Finally, we conclude this article with a discussion and future perspectives. Unlike a comprehensive review that exhaustively looks into every detail of regulations, this review focuses on capturing the salient features of two different sovereign entities and jotting down key policy elements that need to be harmonized. All data used in this article are obtained from literature and publicly accessible data registries.

## 2. Cell and gene therapy R&D and market trend

**Figure 1:**
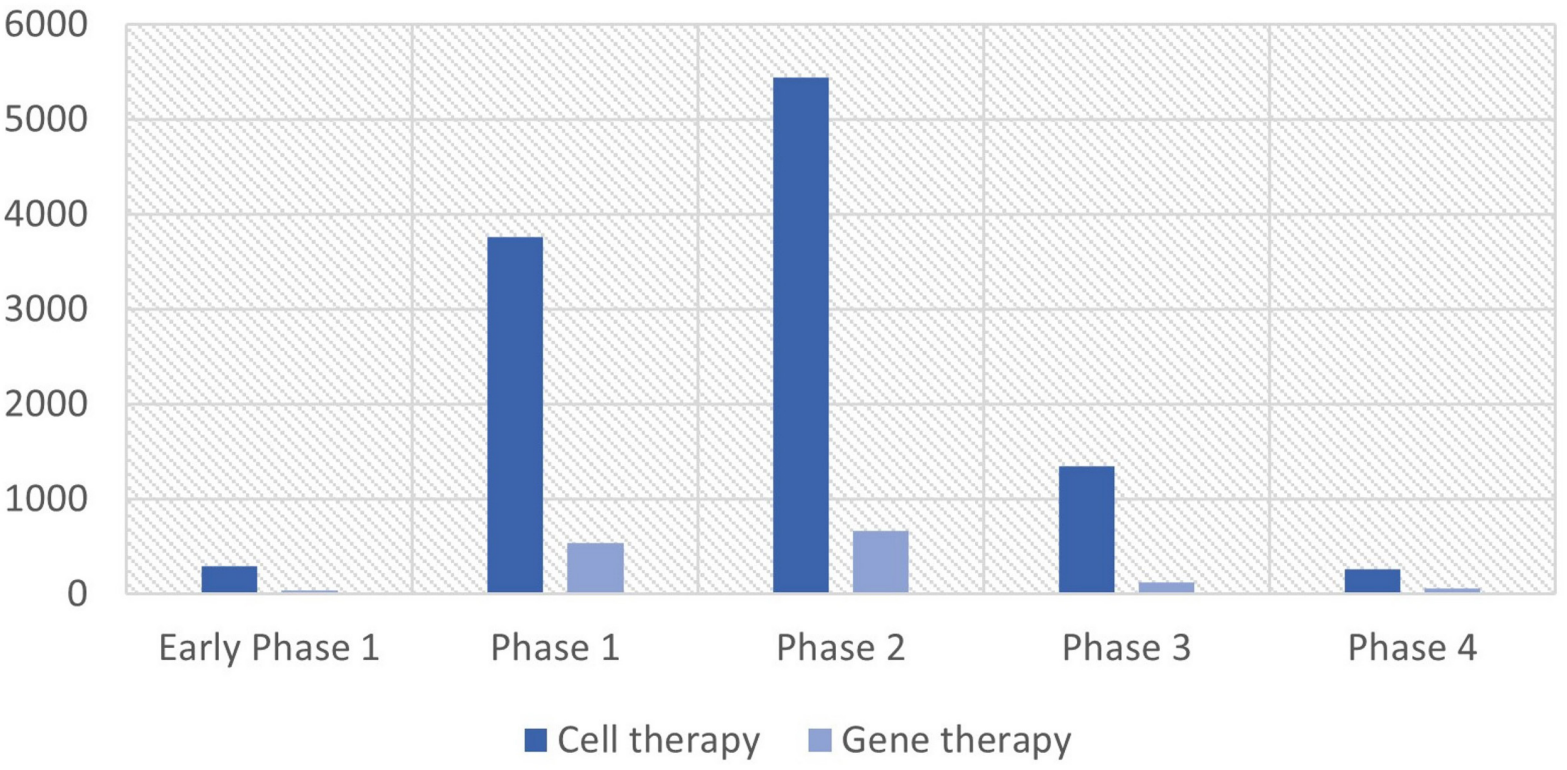
Cell and gene therapy clinical trials by phase. Source: NIH's ClinicalTrials.gov (as of August 2022). The unit of the Y-axis is the number of clinical trials.

**Figure 2:**
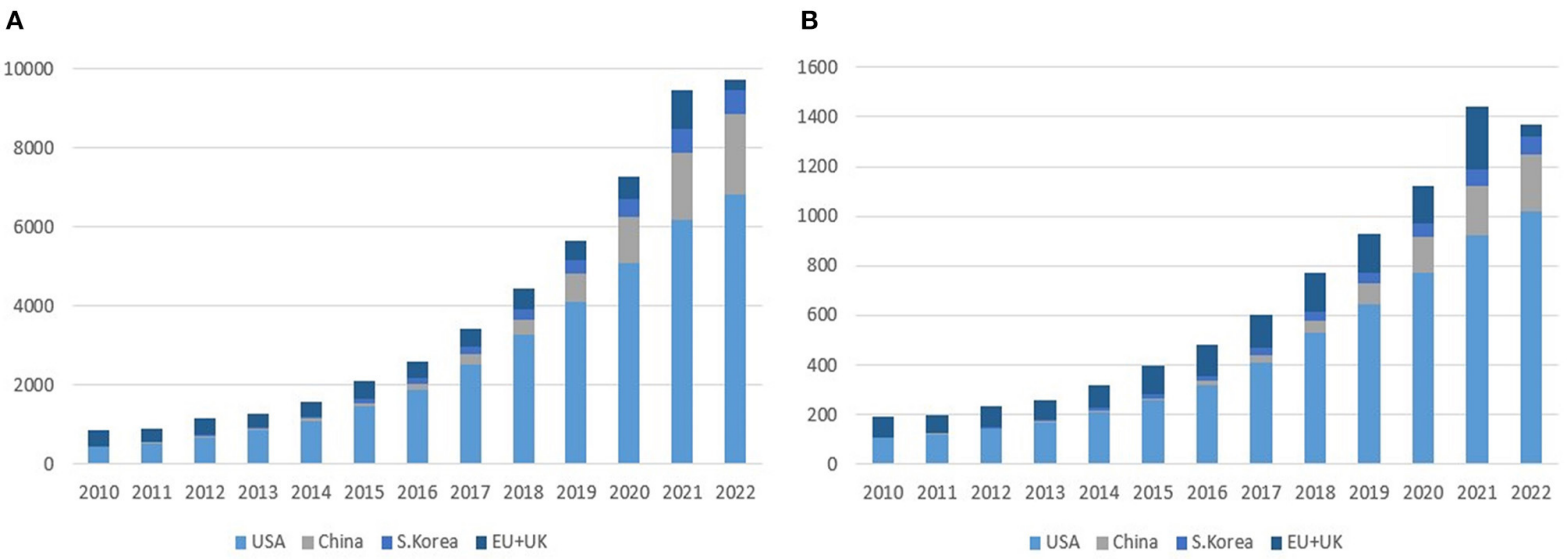
Cell and gene therapy clinical trials by region (2010–2022). **(A)** Cell therapy. **(B)** Gene therapy. Source: NIH's ClinicalTrials.gov, EU's ClinicalTrialsRegister.eu (as of August 2022). The unit of the Y-axis is the number of clinical trials.

As of July 2022, there are 59 cell therapies and 37 gene therapies that include 18 RNA therapies approved worldwide (3). The segregation of cell and gene therapy clinical trials into each clinical phase indicates that a majority of currently active clinical trials are at their earlier clinical stages as of August 2022, especially phases 1 and 2, implying that a wide array of pipelines will reach the market after 3–7 years (Figure 1). Between 2010 and 2022, the total number of cell and gene therapy clinical trials has steadily increased worldwide at a compound annual growth rate of 20–25%. The breakdown of cell therapy clinical trials by countries shows that the EU, the UK, and the US have taken a dominant position in the early 2010s by conducting more than 90% of total cell therapy trials worldwide. However, the predominance of the EU has abated as China sizes up its presence at a compound annual growth rate of 55% over the same time span, ultimately reaching 21% of worldwide pipelines in 2022 (Figure 2). During the same period, the US has beefed up its presence, going from holding a dominant position of 50% of the whole pipeline in 2010 to surpassing 70% by 2022. The proportion of South Korea has increased slowly, reaching 6.4% in 2022 from a mere 1.5% in 2010. Similarly, as the EU, the UK, and the US take up a significant portion of the whole gene therapy pipelines, China's rise becomes more conspicuous taking up to 17% of total pipelines as of 2022.

Research and development in cell and gene therapy is currently on the path of honing their efficacy and safety by delving further into underlying genetic mechanisms and devising ways to tackle adverse immunological responses (Table 1). A significant portion of clinical trials are “*bioparallels*” that are designed by applying successful platforms or methods to new entities: e.g., a chimeric antigen receptor (CAR)-T therapy that uses the same platform and antigen but with a different epitope targeted (9). License deals of CGT pipelines and ancillary technologies take up a significant portion of the market (10). Cell therapies mostly focus on immune cells and stem cells; CAR-T therapies and natural killer (NK) therapies take up most of the immune cell pipelines currently, which now extends to a vast catalog of new modalities, such as gamma delta (γδ) T-cell therapy, CAR-Treg therapy, CAR-M therapy, T cell receptor (TCR)-based therapy, and T-cell antigen coupler (TAC) therapy. The gene therapy market has seen the growth of RNA therapy and adeno-associated virus (AAV) vector-based gene therapy in recent years, and a series of next-generation gene editing platforms being upgraded are geared toward sharpening therapies' specificity, and thus efficacy. The state-of-the-art genome sequencing and single-cell sequencing technologies have spawned a string of personalized genome therapy pipelines.

**Table 1:** Major research and development themes of cell and gene therapies (4–8).

	**Research theme**		**Research Topic**
Cell therapy	Cell differentiation and cell culture		- Research on cell/stem cell differentiation and cell culture to enhance therapy's efficacy and safety - Search for stable biomarkers such as MSC indicating the progress of stem cell differentiation - Research on the differentiation mechanism of iPSC and its biomarkers
	Allogenic/xenogenic cell therapy		- Research on ways to tackle immunological responses
	Immune cell therapy	CAR-T therapy	- Indication: blood cancer ⇒ solid cancer, autoimmune diseases (*systemic lupus* erythematosus), cardiometabolic diseases (type 1 diabetes), fibrosis, cellular senescence, infectious diseases (HIV, hepatitis C, tuberculosis, and invasive aspergillosis) - Research to tackle immune escape, immunological response, fratricide, and cardiotoxicity - Targeting new antigens other than CD19, BCMA - Double, triple-antigen targeting and dual epitope targeting - From *ex vivo* autologous CAR-T to off-the-shelf Allogenic CAR-T: *in-vivo* engineering - On-and-off switches to control cell activity in an attempt to mitigate toxicity stemming from T-cell hyperactivation
		New modalities	- Innate immune cell therapy (NK cell therapy, CAR-Macrophages (*CAR*-*M*) therapy) - Gamma Delta (γδ) T-cell, CAR-Treg, TCR-T, TCR-CAR-NK therapies
	Synthetic biology		- Research on synthetic gene circuits to enhance therapies' specificity and penetration
	Exosome		- Mass production techniques to separate highly-pure exosome
Gene therapy	Viral/non-viral vector		- Research on new viral vectors, trade-offs between different viral vectors on safety, efficacy, and immunogenicity - Engineering viral vectors 1. to deliver to specific cells or tissues, 2. to induce long-lasting immunogenicity by touching on the response mechanism of antibody and T cell or by incorporating an adjuvant/gene 3. to optimize transgene expression by delivering multiple antigens at a time - Standardizing procedures to make sure transfection efficiency and consistency
	Gene-editing technology		- Research on off-target genome editing
	RNA therapy		- Indication: vaccine for infectious disease ⇒ cancer vaccine - Issues with cold chain delivery - New modalities of Non-coding RNAi therapy: small interfering RNA (siRNA), micro RNA (miRNA), circular RNA (circRNA) - ASO therapy: research on drug delivery platform (relatively large molecule size inhabits intercellular or inter-tissue delivery and oral administration)
Manufacturing Technology	- Technologies to scale up and scale out a whole manufacturing process, standardizing into scalable manufacturing platforms - The real-time tracking of the manufacturing process at the cellular and molecular levels: *in-situ* (in-line) and at-line monitoring - Continuous manufacturing process - Distributed manufacturing, Point-Of-Care (POC) production platforms		

ASO, antisense oligonucleotide; CAR, chimeric antigen receptor; CGT, cell and gene therapy; iPSC, induced pluripotent stem cell; MSC, mesenchymal stem cell; NK, the natural killer; TCR, T-cell receptor.

While most of the currently available pipelines target cancer, rare disease, and severe disease, once it gets through commercialization strategies to stand on its own feet, the realm of CGTs will ultimately extend to personalized therapies and, then finally, to common diseases (11).

On the manufacturing side, firms are working on developing manufacturing platforms that will streamline the whole manufacturing process in a compact way: e.g., manufacturing technologies for off-the-shelf cell and gene therapies are pitched for the standardization and scale-up of the manufacturing process.

### 2.1. South Korea market

South Korea's Ministry of Food and Drug Safety (MFDS) has approved 15 cell therapies and three gene therapies (12). All 15 cell therapies are provided by Korean firms and all three gene therapies are imported medicines. So far, one cell therapy voluntarily opted out of the market due to poor sales records. Most cell therapies are non-genetically modified ones for skin wounds and burns. Meanwhile, three cell therapies are mesenchymal stem cell (MSC) therapies, the earliest of which was approved in 2005. In 2021, GC Cell, a subsidiary of Green Cross Biopharma, was granted approval for ImmuCell-LC, an adjuvant cancer immunotherapy.

**Figure 3:**
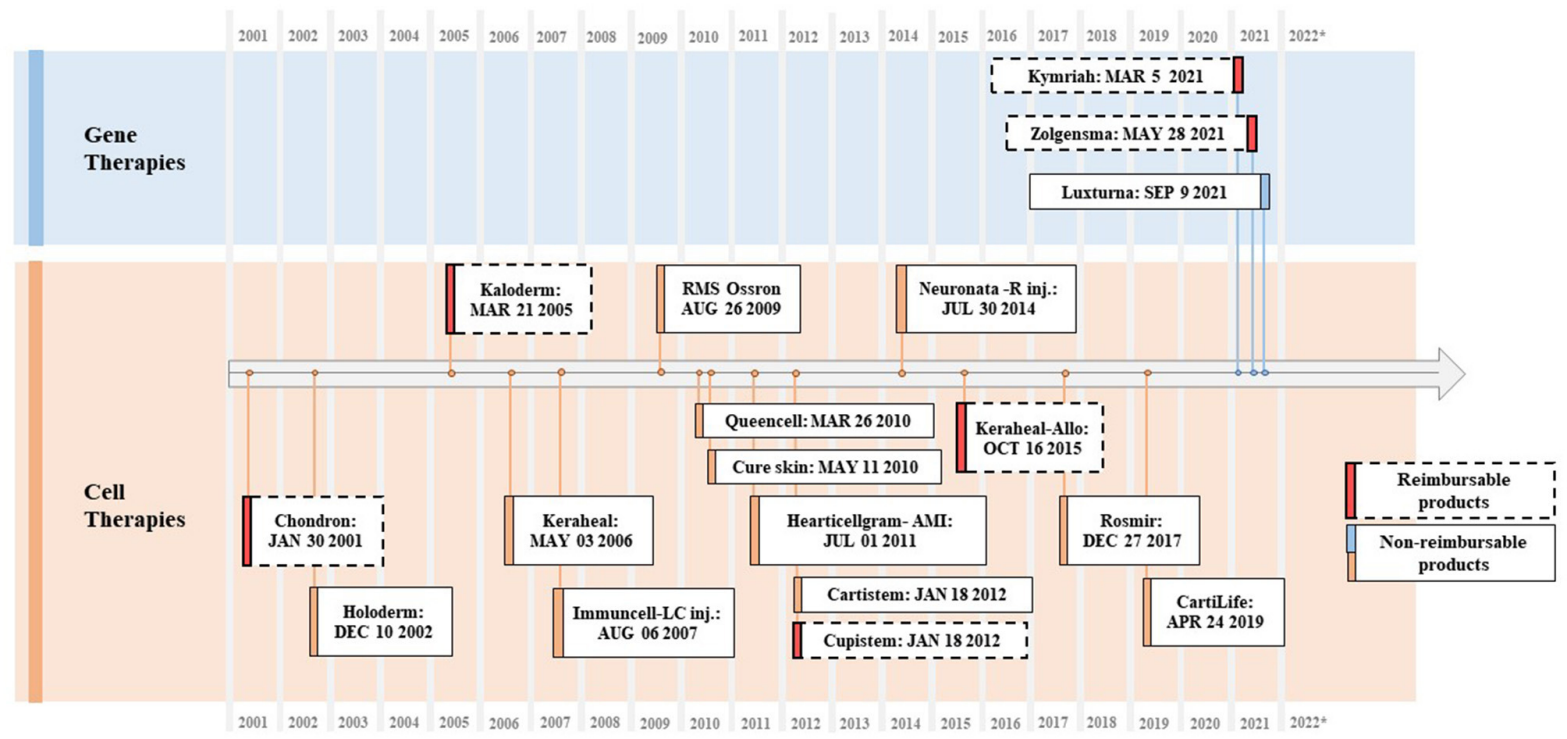
Approved CGTs in South Korea (2001–2022). Source: Ministry of Food and Drug Safety, Health Insurance Review and Assessment Service.

It seems that there are a few years gaps on average for foreign cell and gene therapies that have entered advanced markets such as the US and/or Europe to reach the Korean market finally. Most of the time, developers attempt to try either the US or European market first, considering its market size, pricing mechanism, and advanced regulatory system and, once they proceed to enter, it can take years to pass through all stages of the South Korean's regulatory hurdles. For example, Kymriah, the first kind of CAR-T therapy, was approved by the US Food and Drug Administration (FDA) in August 2017 for B-cell acute lymphoblastic leukemia (ALL) and diffuse large B-cell lymphoma (DLBCL) and received accelerated approval later in May 2022 for follicular lymphoma (FL). However, the MFDS passed Kymriah as late as March 2021 for ALL and DLBCL (Figure 3).

Amid pipelines in progress, 15 of them target non-small cell lung cancer, with five and three targeting non-Hodgkin lymphoma and DLBCL, respectively (13). The technological spectrum and platforms of Korean firms have progressed to include a wide catalog of NK cell therapy (GC Cell Co., NKMAX Co.), RNAi and siRNA gene therapy (mCureX Co., OliX Co.), and CAR-T and CAR-M therapy (AbClon Co., Eutilex Co., JW CreaGene Co., OncoInsight Co.). ToolGen Co., which claimed to have developed the first kind of CRISPR/Cas9 system applied to the eukaryotic cell and currently is in a patent dispute with the University of California, Berkeley and the Broad Institute of MIT, is actively collaborating with other biotechnologies companies to develop CGTs based on its CRISPR/Cas9 platform, with its pipelines spanning CAR-T and mRNA platform-based therapies. G+FLAS Life Science Co. and GenKOre Co. are also frontline developers of the CRISPR-based platform. Most of the frontrunners are small and medium-sized biotech companies. However, increasingly larger pharmaceuticals, such as Chong Kun Dang Co. and Samsung Biologics Co., are plunging into this newer market, looking for M&A (mergers and acquisitions), joint R&D, and licensing opportunities.

Reyon Pharmaceutical Co., SK Bioscience Co., GC Cell Co., Daewoong Pharmaceutical Co., Lotte Biologics Co., and MEDIPOST Co. have entered the cell and gene therapy contract development and manufacturing organization (CDMO) market. Furthermore, a mass production system is expected to be set up between 2023 and 2025.

### 2.2. The EU market

The Committee for Advanced Therapies (CAT) of the European Medicines Agency (EMA) is in charge of classifying advanced therapy medicinal products (ATMPs), which are primarily classified into three categories: gene therapy medicinal products (GTMPs), somatic cell therapy medicinal products (sCTMPs), and tissue-engineered product (TEP). As of October 2022, the EMA had approved three cell therapies, 16 gene therapies, and four tissue-engineered products; five out of 23 withdrew marketing authorizations (MAs); and two did not renew MAs and opted to exit the EU market (14).

**Figure 4:**
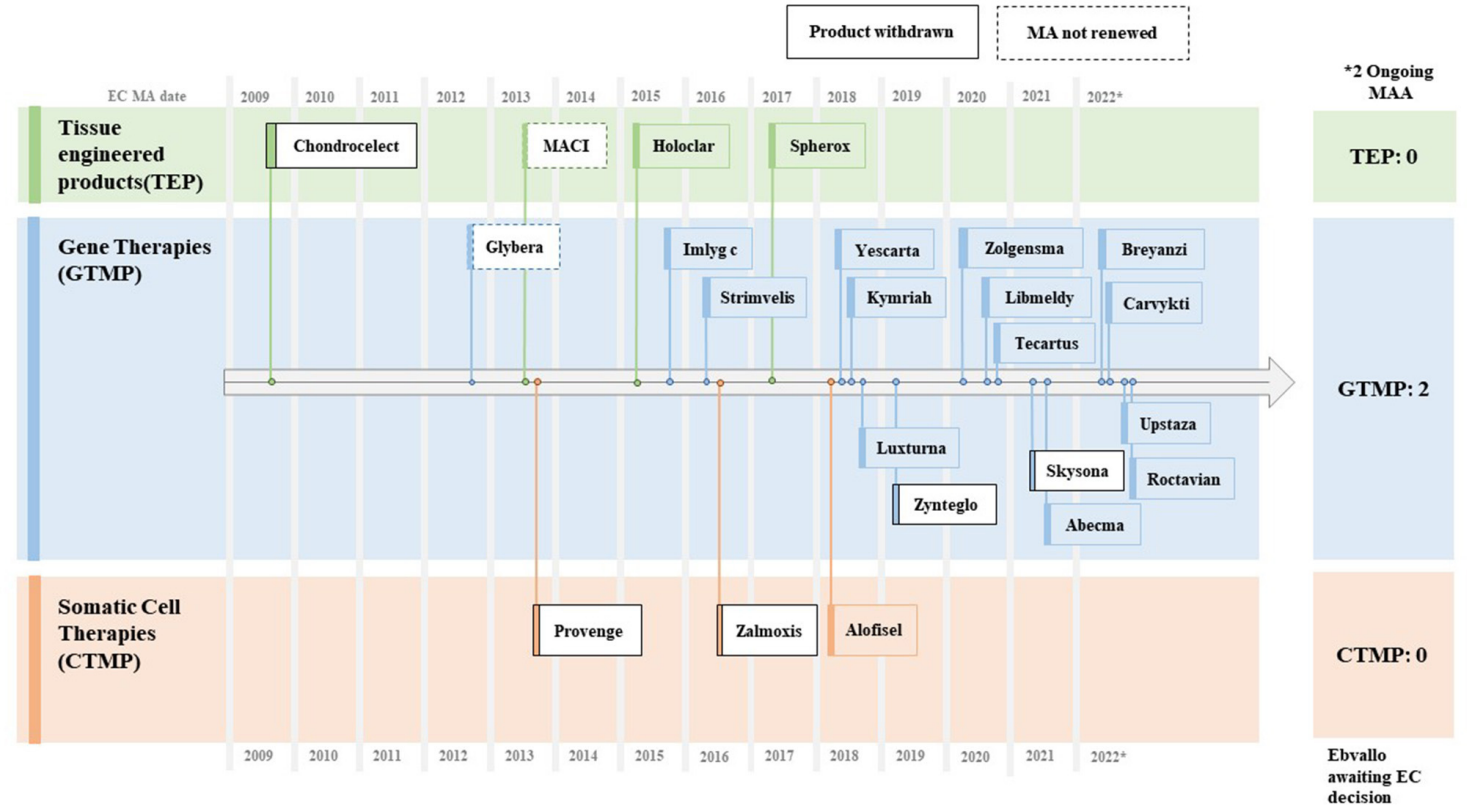
Approved CGTs in EU (~2009–2022). Source: EMA CAT Quarterly Highlights and Approved ATMPs (Oct. 2022).

Among the reasons for withdrawals were commercial failures owing to steep pricing (e.g., Provenge^®^, ChondroCelect^®^, Glybera^®^, and MACI^®^), deadlocks in the process of reimbursement negotiations (e.g., Skysona^®^ and Zynteglo^®^), and unfavorable confirmatory clinical results after initial conditional approvals (e.g., Zalmoxis^®^). Later, Skysona^®^ and MACI^®^ managed to get clearance from the FDA with a gap of 1–3 years (Figure 4).

Approved indications cover hematological cancers, spinal muscular atrophy, beta-thalassemia, inherited retinal disease, cartilage disease, aromatic L-amino acid decarboxylase (AADC) deficiency, and hemophilia A. Development pipelines extend to solid tumors, hemophilia B, diabetes, cardiovascular disorders, neurological disorders, sickle cell disease, neoplasms, and pancreatic cancer (15). Approximately 20–30 new pipelines are expected to be put forth for ATMP approvals in the next 5 years (16). By modalities, CAR-T and NK cell therapies and gene therapies using gene editing platforms have seen the most uptick during the last couple of years (15).

The UK, the Netherlands, Italy, Germany, France, Sweden, Belgium, and Switzerland are considered the hotspots for settlement by cell and gene therapy biotechnology companies. From 2022 to 2030, Germany, France, Italy, and Spain are expected to lead the ATMP service market in the EU, which can be segmented into contract research organization (CRO), contract manufacturing organization (CMO), quality, and logistics (17, 18).

## 3. Government regulations and industrial policies for cell and gene therapy

A slew of money has flocked to the cell and gene therapy market over the past few years, owing to sufficiently matured bioengineering technologies reaching the platformable commercialization stage, bearing fruits in the form of abundant clinical studies in the field. Newer modalities have induced a string of regulatory and legislative tasks, such as guidelines and legislative rules to systematically regulate novel pharmaceutical products. Accordingly, another layer of legislation and guidelines tailored for CGTs has been introduced, which is expected to evolve on par with technological progress.

### 3.1. Regulations and industrial policies of the South Korean government

The South Korean government enacted the “Act on the Safety and Support for Advanced Regenerative Medicine and Advanced Biological Products” in August 2019, which has been effective since August 2020. Before introducing the act, CGTs were classified as usual biopharmaceuticals. Advanced regenerative medicine pertains to medical treatment, and advanced biological products (ABPs) can be classified into cell therapy, gene therapy, tissue engineering products, and advanced biological combination products. The act aims to (i) prepare a safety management system for advanced biopharmaceuticals that details safety protocols and standards, (ii) plan regulatory science and a long-term follow-up system, and (iii) set up the details of expedited and conditional approval procedures (19, 20).

Under this act, the Advanced Regenerative Medicine and Advanced Biological Products Policy Review Committee (i) formulates master plans, a kind of R&D policy roadmap, and corresponding action plans that are usually published yearly and (ii) is in charge of matters regarding the classification of advanced regenerative medicines, approval of clinical research, operation of long-term follow-ups, designation of advanced regenerative medicine implementation institutions that conduct clinical studies, and advanced regenerative medicine cell processing establishments. The committee formulated “the first 5-year plan for the Advanced Regenerative Medicine and Advanced Biological Products (2021–2025)” in January 2021; it publishes yearly action plans in January.

Clinical researches of advanced regenerative medicine is a legal pathway that grants patients access to life-saving advanced regenerative medicines for non-commercial purposes. Clinical researches have to get approved by the committee beforehand, whose track consists of high and moderate to low-risk clinical studies (Table 2).

**Table 2:** Major regulatory framework for cell and gene therapies in South Korea (2, 5, 21–24).

**Sector**	**Details**
Clinical researches	- Clinical researches of advanced regenerative medicine should be approved by the Advanced Regenerative Medicine and Advanced Biological Products Policy Review Committee in advance and only authorized medical practitioners at authorized hospitals can be in charge of treatment. - Consists of two tracks of high and moderate to low-risk clinical research. High-risk clinical studies have to pass through an additional assessment stage by MFDS. - Therapeutic usage of foreign investigational drugs will be conditionally allowed (announced in August 2022).
Drug ingredients	- Eligibility guidelines for cell banks and cell donors published - Firms handling cell and gene therapy ingredients prerequired to get certified and certification has to be renewed every 3 years.
Manufacturing	- GMP guidelines for advanced biological products published and to be modified in accordance with newly published PIC/S standards.
Approval	- Rare and severe disease therapies eligible for conditional approval, priority screening, and customized examination - GIFT(Global innovative products on the fast track) introduced: An expedited track for innovative drugs is newly launched, with its whole span to be shortened by 1/4 compared to regular tracks (announced in August 2022). Global standards that are not inscribed yet in its domestic version to be made applicable in advance in the cases of innovative drugs
Follow-ups	−30 years follow-up after administration of cell therapies, 15 years for gene therapies, and 5 years for stem-cell therapies - In November 2021, MFDS and KIDS opened up an online platform where doctors licensed for the handling of CGTs and patients can report and track a whole stage of the treatment process. - Ongoing investigations that study COVID-19 vaccines' safety and effectiveness by analyzing RWD/RWE - RWD/RWE to be fed back into the approval, pricing, and reimbursement reassessment process (announced in May 2022)
Pricing and reimbursement	- CEA rules, Pricing-usage linkage, foreign reference prices for expensive drugs to be modified to advocate better access (announced in July 2022): conducting CEA evaluation at the post-marketing stage after CEA exemption imposed, maximum reduction rates to be adjusted upwards in alignment with usage, re-evaluation on the composition of reference countries and real-time updates of reference prices
Infrastructure	- Global International Policy Strategies Taskforce to be launched: actively engaging in international cooperations such as ICH meetings and expanding mutual recognition agreements (MRA) networks, preemptively tackling trade issues such as non-tariff barriers (announced in August 2022)

CGT, cell and gene therapy; CEA, cost-effectiveness analysis; MFDS, The Ministry of Food and Drug Safety; GMP, good manufacturing practice; ICH, International Council for Harmonization; KIDS, Korea Institute of Drug Safety & Risk Management; RWD, real-world data; RWE, real-world evidence.

High-risk clinical researches must undergo an additional approval process by MFDS after the evaluation by the committee. The committee mandates that cell donors go through eligibility tests. Clinical researches are eligible for a 2 year-government subsidy, with high-risk clinical studies eligible up to 0.7 million USD/year, moderate-risk clinical studies for 0.4 million USD/year, and low-risk clinical studies for 0.2 million USD/year.

Advanced biological products are eligible for (i) “conditional approval” whose decision is provisionally based on surrogate endpoint results from phase 2 results with the condition that the results of confirmatory clinical trials (phase 3) are to be submitted later (ii) “priority screening” that can accelerate assessment process, by assigning priorities to those eligible (iii) “customized examination” that preliminarily reviews documents handed in advance according to schedule of developers, not requiring whole packages to be completed at the final submission stage at once. The Advanced Regenerative Medicine and Advanced Biological Products Policy Review Committee designates and manages the eligibility list, which includes advanced biopharmaceuticals for serious diseases without alternatives, rare, life-threatening diseases, and highly infectious diseases. Regulations connecting clinical researches to commercial clinical trials and finally to approval stage are now under study. Patients who are prescribed advanced biological products containing animal organs or cells, those with gene therapy prescriptions, and those with stem-cell therapy prescriptions should be followed up for 30, 15, and 5 years, respectively.

The Ministry of Food and Drug Safety has published and updated guidelines for advanced biological products since as early as 2005. They are not legally binding but reflect the position and interpretation of MFDS on the subjects, like other countries. The list includes guidelines on preclinical evaluations, the design and analysis of clinical trials for conditional approval, assessment standards for specific products, donor eligibility for cell therapy products, good manufacturing practice (GMP), and patient follow-up. MFDS has a tendency of following in the footsteps of the FDA and the International Council for Harmonization (ICH), publishing or revising its own versions of guidelines with at least a year or few years delay considering versions of the FDA or ICH as examples. However, in August 2022, MFDS published the world's first guideline on the quality assessment of mRNA-based gene therapy products in Korea.

The Ministry of Food and Drug Safety and the Korea Institute of Drug Safety and Risk Management govern the follow-up systems. They launched an online platform in November 2021 where doctors are licensed for the handling of ABPs, and patients can report and track an entire treatment process.

In August 2022, MFDS announced “100 Policy Tasks for Food and Drug Regulatory Innovation.” This entire raft of policy tasks is expected to uphold the access of patients to innovative drugs, which are keenly relevant for CGTs as well (21). Most notably, Global Innovative Products on Fast Track will be launched to set up a fast track for innovative products, while fast tracks so far have been mostly open for orphan drugs and anti-cancer drugs by taking into consideration the severities of diseases. MFDS has also announced its policy direction of advancing the application of global assessment and approval standards, such as the ICH guidelines, even if those standards have not yet been incorporated into South Korea's statute or the guidelines of MFDS.

Another issue about CGTs includes allowing the therapeutic uses of investigational drugs that have not undergone clinical trials or clinical studies approval processes in South Korea. The usage of investigational drugs is granted only for therapeutic purposes under the following conditions that : (i) they pass the clinical approval process by their domestic authorities, (ii) there are no approved alternative treatments in the market for the specific indication, and (iii) it is used for treating severe diseases. The new rule is expected to be applicable by December 2023. Finally, the Global Food and Drug Policy Strategy Committee will be assigned to lay out export-supportive systems by providing a comparative analysis of major regulations from foreign authorities and preemptively tackling trade conflicts.

As part of industrial policies, the government runs 51 government-sponsored R&D projects designated for advanced regenerative medicine and advanced biological products, 16 of which are about basic technology, 31 about translational technology, and four about advanced regenerative medicine and biological products (25).

In detail, the Ministry of Trade, Industry, and Energy has planned to embark on a project to support the manufacturing technology development for ABP and boost the overall market ecosystem, with ~461 million dollars earmarked for the project (25). A preliminary feasibility investigation began in March 2022. If passed, the project will be started in 2024. In June 2022, the ministry announced that cell and virus vector manufacturing technologies for advanced biological products, manufacturing technologies of cell culture equipment, and microbial culture medium will be designated as “essential strategy technologies” (22). Thus far, the list of essential strategy technologies primarily includes cutting-edge technologies from the semiconductor, display, electronics, basic chemical, and automotive industries that are deemed critical for industrial value chains. Those listed will receive preferential treatment from government-sponsored projects, laxed regulations on employment and the environment, and tax credits for M&A and capital investment (19).

The small and mid-size enterprise (SME) strategic technology roadmap of the Ministry of SMEs and Startups has designated seven ABP technologies as promising strategic technologies and planned a mid-term target (2022–2026) of technological development as guidelines. Seven technologies include (i) vector technologies for gene therapies, (ii) immune cell therapy technologies, (iii) stem cell technologies, (iv) adult stem cell therapy technologies, (v) efficacy testing technologies for ABPs, (vi) drug delivery technologies using self-assembled nanoparticles, and (vii) peptide-based cancer vaccine technologies using cancer-cell epitopes (26).

In July 2022, the Ministry of Health and Welfare launched the K-Bio Vaccine Fund, the eighth of its mega-funds, aiming to facilitate the development of innovative new drugs and vaccines with a funding pool of 0.3 billion USD (500 billion KRW) (27). The ministry aims to equip its cash-stricken domestic market with ample liquidity so that Korean companies can lead themselves through the final stage of drug approvals, rather than opting for licensing deals in the middle of clinical stages due to the financial burdens of phase 3 clinical trials.

Finally, ministries jointly planned export-supportive strategies in August 2022, which included regulatory exemptions for raw materials entering bonded areas and easier credit conditions, especially for SMEs who lack track records of revenues, by placing more weight on the financial structure of new entrants (28). It will also lay the groundwork for (online) platforms that will facilitate business matches, hopefully resulting in more open innovation opportunities, including licensing deals, M&A, and joint research.

### 3.2. EU regulations and industrial policies

Regulation (EC) No 1394/2007 is the underpinning legal framework for ATMPs, which was introduced in 2007. Subsequently, other regulations and directives have been amended after enacting Regulation (EC) No 1394/2007, whose issues that are relevant to ATMPs include good clinical practice, GMP, pharmacovigilance, and dealings with personal data. EMA mandates that ATMPs have to pass through centralized authorization procedures, not national-level screening. Its validity will be effective for all EU member states once it has passed. EMA irregularly publishes guidelines for ATMPs. The follow-up duration is determined on a case-by-case basis, while patients are usually followed up for 15 years after the administration of gene therapies containing vectors (29).

Support of EMA for ATMP developers is mostly administrative or technical, coupled with snippets of monetary support (Table 3). EMA offers a 65% fee reduction to ATMP developers for scientific advice on technological queries raised during drug development, with additional preferential rates of 90% set for micro, small, and medium-sized enterprises (SMEs) status and 90% for fee reductions for the certification procedure (32). The Innovation Task Force facilitates informal dialogues in earlier stages between EMA and ATMP developers and offers scientific, legal, and regulatory guidance. ATMPs developers are also eligible for (i) accelerated assessments aiming to shorten the entire process of centralized authorization from 210 to 150 days, (ii) Priority Medicine Scheme (PRIME) designation that can expedite the whole development process by providing developers with customized scientific advice during earlier stages, (iii) orphan designation that benefits in the form of incentives, such as market exclusivity, fee reduction, and scientific advice, and (iv) SMEs status that makes firms eligible for a range of assistance and guidance. Between 2016 and 2022, 110 ATMP pipelines filed for PRIME designation, but only less than half of them, 48, were assigned the designation. A total of 535 ATMP pipelines have sought scientific advice since 2009 (14).

**Table 3:** Major regulatory framework for cell and gene therapies in EU (5, 30, 31).

**Sector**		**Details**
Regulatory support		- At earlier stages, the innovation task force (ITF) offers scientific, legal, and regulatory guidance and facilitates dialogue channels between EMA and ATMP developers. EMA offers Scientific Advice throughout the whole stages of clinical trials. - A 65% fee reduction for scientific advice, with additional preferential rates of 90% set for SMEs, and 90% of fee reductions for the certification procedure - A pilot program to help non-profit academics with regulatory guidance and support has launched in Sep 2022.
Approval		- ATMP developers are eligible for accelerated assessments/PRIME designation/orphan designation/SME status.
Follow-ups		- Follow-up durations are to be determined on a case-by-case basis, while patients are usually followed up by up to 15 years after the administration of gene therapies containing vectors.
Pricing and reimbursement	RWD	- France, Italy, Spain, and Germany utilize RWD to test safety and effectiveness and publish cost-effectiveness analysis reports. Outcome-based payment or outcome-based reimbursement programs are in place. - EMA plans to build a pan-European RWD registry. - Hospital exemption is a regulated pathway that grants access to unauthorized ATMPs under restricted conditions, mostly in the case of unmet medical needs. Unlike the centralized authorization procedure, specific implementation rules are set by individual national authorities and vary across the EU.
Infrastructure		- EU-funded European Consortium for Communicating Gene and Cell Therapy Information (EuroGCT) was launched in Feb 2021.

ATMP, advanced therapy medicinal products; EMA, European Medicines Agency; SME, small and mid-size enterprises; PRIME, Priority Medicine Scheme; RWD, real-world data.

As an initiative to spur ATMP development, the EU's Horizon 2020—the EU's funding program geared for encouraging research and innovation—has funded the European Consortium for Communicating Gene and Cell Therapy Information (EuroGCT) since February 2021 with an initial plan of 5 years span (33). This consortium connects 49 research institutions and universities across Europe and it aims to keep stakeholders informed with the latest scientific, legal, ethical, and societal knowledge regarding CGT development. In September 2022, EMA launched a pilot program that provided non-profit institutions and academics involved in developing ATMPs with regulatory support, fee reductions, and waivers (34). A CAR-T pipeline (ARI-0001) has been picked as the first recipient and EMA is expected to guide them through regulatory processes.

## 4. Pricing and reimbursement policy for CGTs in South Korea and the EU

The P&R policies of innovative CGTs hinge on multifaceted elements that include the accessibility of a patient to treatment, monetary compensation for R&D expenses of pharmaceutical companies (developers), and budget impact on patients and national insurance (35–38). Generally, CGTs target severe and rare diseases for which alternative treatments are not yet out on the market, and the dosing cost per unit is considerably high due to R&D costs and a relatively smaller patient population. In addition, clinical uncertainties coming from pharmaco-characteristics of CGTs require follow-ups of several years, complicating the P&R appraisal process (39, 40).

The core tenet of P&R policies is to obtain a balance between securing the accessibility of patients to innovative treatment, optimal management of a limited budget, and clinical uncertainties of pricey one-off drugs, whose analysis can be substantiated through the cost-effectiveness analysis (CEA). A tenacious voice that demands the incorporation of broader societal benefits into the P&R appraisal system has emerged and the P&R policies for innovative CGTs have evolved to strike a balance among the aforementioned conflicting values. However, proving the cost-effectiveness of one-off CGT is not straightforward because there is a risk that the cost of the treatment can end up as a social loss if the patient's outcome is not satisfactory after the treatment cost is paid out upfront (41).

Accordingly, outcome-based managed entry agreements (MEAs) have been introduced to tackle clinical uncertainties (42, 43); their design mandates drug developers and insurance agencies to contribute in the event of unsatisfactory outcomes, along with other budget-constrained MEA modes that cap the total expenditure. Especially, outcome-based MEAs are often adopted as a major policy tool to improve patients' access to innovative CGTs, with pharmaceutical companies and authorities both rightfully bearing some risks arising from exorbitant prices and clinical uncertainties (42). A combination of two separate modes can be applied as well. Mostly, the details of MEA are confidentially negotiated and remain publicly undisclosed. It draws criticism that end results are not always socially optimal, leaving the chance of Pareto improvement untapped in the end. In addition, ex-post payment modes conditional on treatment results have also been introduced (44, 45). Other miscellaneous measures can work in tandem: such as authorization for temporary use (ATU) (France) and new examination and treatment method (NUB1) (Germany) introduced to hasten the overall R&R process for innovative drugs and special grants for innovative medicines (Italy).

Global standards of P&R systems are closely intertwined with each other, and the long-term trajectory has progressed to harmonize standards among closely connected markets (46–49). The South Korean HTA system refers to the prices of CGTs in the following advanced seven (A7) countries that include the EU5: the USA, Japan, France, Italy, Switzerland, Germany, and the UK. On the contrary, other countries such as China also use South Korean drug prices as one of their reference prices. Therefore, studies on the P&R mechanisms of advanced countries hint at a possible trajectory that other countries can take in the near future.

### 4.1. P&R policy for innovative CGTs in South Korea

The P&R appraisal process for CGTs in South Korea adopts the same standard process as other medicines, and only drugs that have been proven cost-effective are positively listed in the reimbursable drug formulary (50, 51). Cost-effectiveness must be proven through a PE study, or a PE waiver must be applied to list expensive CGTs without alternatives in South Korea (51). However, listing CGTs through the PE study process is practically difficult due to the aforementioned characteristics of CGTs, the small patient population, and clinical uncertainties. Similarly, in South Korea, collecting sufficient clinical evidence for clinical necessities, such as orphan drugs, anti-cancer drugs, and severe life-threatening diseases, is challenging due to the small pool of patients.

Hence, the Korean government has given leeway with a wide range of incremental cost-effectiveness ratio (ICER) thresholds [maximum 50 million/quality-adjusted life year (QALY)], particulars of RSA, and exemption rules for CEA and pharmacoeconomic (PE) waivers to improve patients' access to innovative new drugs (52–55). For the cases of orphan drugs, anticancer drugs, and severe life-threatening diseases, the CEA can be exempted, and the expenditure-capped RSA becomes effective and the prices of A7 countries are referred to as reference prices (Table 4). In the case of CGT for which CEA is not possible, RSA, whether expenditure-capped or outcome-based type, becomes effective, and A7 reference prices are referred to (51).

**Table 4:** Criteria for cost-effectiveness analysis exemption in South Korea (52, 55).

**Criteria**	**Reference price**	**Risk sharing agreement**
Meet all the following criteria 1. Drugs for anticancer or rare disease treatments in the case of i) no alternatives out in the market or ii) no clinically equivalent drugs or treatments, and for treating serious life-threatening conditions 2. Drugs approved with single-arm clinical trials or conditionally approved based on phase 2 clinical trial results, most of which are restricted by a smaller population of patients available for clinical trials 3. Listed in at least three of A7 countries	The lowest of A7 adjusted prices	Should be expenditure cap. type

A7 countries refer to advanced seven counties (the USA, the UK, Italy, Germany, Japan, Switzerland, and France).

The mechanism of RSA is designed to have all stakeholders, such as drug developers and insurance authorities, evenly held for clinical uncertainties to hedge against adverse outcomes. In 2013, South Korea introduced the RSA, which can be largely bracketed into outcome-based and budget-constrained modes—either expenditure-capped or per-patient-cost capped; a mix of these modes can be negotiated throughout (52). Among these, the design of outcome-based RSA makes drug developers and insurance authorities partially responsible for refunds upon unsuccessful treatment outcomes to partially compensate patients for the risk of taking clinically unapproved medicines.

A total of 15 cell therapy products have received MA in South Korea, and six of them have been listed in the drug reimbursement formulary. Of the six products, two were listed before implementing the current P&R system, and four were listed under the current system. Three gene therapy products received MA, and two of them were reimbursed. A PE waiver was applied to two reimbursed products, upon which outcome-based RSA was applied; their prices were based on the lowest price among the adjusted list prices of A7 countries (see Figure 3 and Table 5).

**Table 5:** Reimbursed cell and gene therapies in South Korea.

**Product**	**Indication**	**Relatives**	**PE waiver**	**RSA**	**Reference price**	**List Price (USD)**	**Reimbursement date**
Chondron	Knee cartilage defect	NA	NA	NA	NA	5,618/imp	Mar 8, 2002
Kaloderm	Skin keratinocytes	NA	NA	NA	NA	255/dose	Mar 21, 2005
Cupistem	Fistula treatment due to Crohn's disease	Infliximab	NA	NA	IRP	9,861/dose	Jan 1, 2014
Keraheal-Allo	Deep second-degree burns	Kaloderm	NA	NA	IRP	484/syringe	Oct 1, 2016
Kymriah	ALL (B-cell acute lymphoblastic leukemia), DLBCL (diffuse large B-cell lymphoma)	NA	√	Expenditure cap/Refund/ Outcome based Refund	ERP (Japan)	263,186/syringe	Apr 1, 2022
Zolgensma	SMA (spinal muscular atrophy)	NA	√	Expenditure cap/Refund/Outcome based Refund	ERP (Japan)	1,448,630/syringe	Aug 1, 2022

P&R, pricing and reimbursement; RSA, risk sharing agreement; WAP, weighted average price of relatives; IRP, internal reference price; PE, pharmacoeconomic study;ERP, external reference price.

The exchange rate used to convert KRW into USD is fixed at 1 USD = 1,368 KRW (22.08.31).

Source: Ministry of Food and Drug Safety, Health Insurance Review and Assessment Service. √, Kymriah was evaluated by PE waiver.

In July 2022, the Korean government announced a set of schemes to improve patients' access to highly priced but clinically effective drugs, where it also newly defined “expensive drug” as a one-shot therapy with an annual dosing cost surpassing 0.3 billion KRW (0.2 million USD) per patient or a single ingredient product with claims to reach more than 0.2 million USD per year (2); It includes accelerating the approval and P&R evaluation processes, expanding the application of RSA, engaging actively in international cooperation for regulatory harmonization, and establishing a pre-approval system that appraises reimbursement decisions on expensive new drugs on a case-by-case basis. The government also announced shortening the entire P&R procedure for severe life-threatening diseases by 60 days, from a total of 210 to 150 days. Especially for severe life-threatening diseases or when alternative medicines are not yet available on the market for the indication, it plans to simultaneously embark on three evaluation processes—approval, pricing, and reimbursement—to speed up the entire procedure.

### 4.2. P&R policy for innovative CGTs in EU countries

Unlike the approval process, which must go through a centralized authorization process by EMA, P&R falls under the control of the governance of each member state. On average, the P&R appraisal process takes the shortest time span of 120 days in Germany, with Switzerland (166 days), Denmark (169 days), and Italy (418 days) trailing behind. European countries, including the UK (335 days) and others, take 504 days on average (56, 57).

As we stated earlier, the P&R decisions of the EU5 bear huge significance to South Korea since the EU5 is part of its reference countries. Most of the CGT products that are reimbursed in the EU5 are processed through MEAs, which can be broadly grouped into two kinds: outcome-based MEAs and budget-contingent MEAs (39, 40, 43, 47, 58). All expensive one-shot gene therapy products (Kymriah^®^, Yescarta^®^, Luxturna^®^, and Zolgensma^®^) are assigned to the outcome-based MEAs (Table 6). It is noteworthy that the list prices of CAR-T cell therapies in EU5 countries are conspicuously aligned, which is presumed to be largely driven by the external reference pricing (ERP) mandating authorities to refer to mutual prices across neighboring countries (42).

**Table 6:** Aligned pricing of the selected cell and gene therapies across five EU countries (39, 40, 47, 59).

**ATMP**	**market Authorization**	**Country**									
		**Italy**		**UK**		**Germany**		**Spain**		**France**	
		**Reimbursement mechanism**	**Price**	**Reimbursement mechanism**	**Price**	**Reimbursement mechanism**	**Price**	**Reimbursement mechanism**	**Price**	**Reimbursement mechanism**	**Price, discounts**
Kymriah	Aug 22, 2018	Outcome-based reimbursement (payment by result)	320,000 €	CED	282,000 £ 313,766 €	CED, outcome-based reimbursement (rebate by result)	320,000 €	Outcome-based reimbursement (payment by result)	320,000 €	CED	320,000 €
Yescarta	Aug 23, 2018	Outcome-based reimbursement (payment by result)	327,000 €	CED	280,451 £ 317,000 €	CED, outcome-based reimbursement (rebate by result)	327,000 €	Outcome-based reimbursement (payment by result)	327,000 €	CED	327,000€
Luxturna	Nov 22, 2018	Budget caps (21.6 Min €/24 months)	360,000 €	–	341,000 €	–	345,000 €	Ceiling cap.	345,000 €–	–	290,000€
Zolgensma	May 18, 2020	Outcome-based reimbursement (payment by result)	3,210,000 €		1,795,000 £	Outcome-based reimbursement (rebate by result)	2,325,000€	–	–		-

CED, coverage with evidence development.

In regards to the details of individual countries' major P&R policies, especially in the EU5 countries, ATMPs are all classified as medicines, and the traditional appraisal P&R process is applied in the same way as other medicinal products, except in Germany (47). Unlike other countries, Germany evaluates ATMPs by classifying them either as medicine (AMNOC process) or medical procedure (PEI process) (47). EU5 countries have not put aside a track distinctively designated for CGTs. However, they operate various systems as part of an effort to improve access to innovative CGTs (Table 7). The UK has launched the highly specialized technologies program (HSTP), where CGTs for ultra-rare diseases are evaluated based on a weighted ICER threshold (maximum 300,000 GBP) (60, 61). The HSTP evaluates CEA by projecting over a longer time span and technological innovativeness beyond health benefits. Strimvelis^®^, Luxturna^®^, and Zolgensma^®^ are those products that have undergone the HSTP (61, 62). In addition, Cancer Drug Fund has been launched to smooth the reimbursement process of anticancer drugs. CAR-T cell therapies, such as Kymriah^®^ and Yescarta^®^, are covered by this system (63).

**Table 7:** Schemes to improve the accessibility of cell and gene therapies across EU countries.

**Country**	**Scheme**	**Cell and gene therapies**
Italy	Innovative medicine	Strimvelis, Kymriah, Yescarta, Luxterna, Zolgensma
UK	HST (Highly Specialized Technologies Programm)	Strimvelis, Luxterna, Zolgensma
	CDF (Cancer Drug Fund)	Kymriah, Yescarta
Germany	NUB1	Imlygic, Zalmoxis, Alofisel, Kymriah, Yescarta, Luxterna
France	ATU	Kymriah, Yescarta, Luxterna, Zolgensma

NUB, Neue Untersuchungs-und Behandlungsmethoden (new examination and treatment method); ATU, Autorisation Temporaired'Utilisation (authorization for temporary use).

The diagnosis-related group (DRG) system also offers an extra-cost coverage benefit. In Germany, extra-budgetary reimbursement is available for CGTs with operational DRG, once given the status of NUB1 (47). Italy provides reimbursement benefits to CGTs certified as innovative medicine through a specialized fund. Strimvelis^®^, Kymriah^®^, Yescarta^®^, Luxturna^®^, and Zolgensma^®^ have been granted innovative status (47, 64).

As for policy initiatives that grant earlier access, some European countries have provisional early access available while making P&R decisions. In France, firms can freely set interim prices under ATU before approving the formal MA decisions, in which pricing is constrained by an annual spending cap, and price discrepancies are supposed to settle ex-post (40, 65, 66). CGTs, such as Kymriah^®^, Yescarta^®^, and Zolgensma^®^, have benefited from this system (67). France also guarantees earlier access to non-innovative drugs for unmet medical needs (67). Germany also grants firms a provisional 12-month period to set their prices freely before officially taking P&R decisions (40, 68). Furthermore, Italy also set up routes for early access to the non-repetitive use of advanced therapies and law 94/98 lays the legal background to try for other indications for which approvals have not been granted (69).

## 5. Discussion

As of now, regulations and rules on pricing and reimbursement for cell and gene therapies are not pretty much aligned worldwide, which acts like non-tariff barriers to trade. Practically, those regulatory hurdles weigh down technological advances, stifling the entrance of innovative medicines. For instance, on average, South Koreans have access to only 35% of new drugs; however, Americans and Germans have access to 87 and 63% of novel drugs, respectively (70). In South Korea, delays in the reimbursement process play a bigger role in disturbing new entrants to its market than those in the approval process, with five out of 10 new drugs stuck in the reimbursement process for more than 3–4 years based on the approved drug data compiled between 2007 and 2019 (71). Similarly, Skysona^®^ and Zynteglo^®^ have been withdrawn from the European market. However, it managed to get through the reimbursement process in the US 1–2 years later.

The ultimate direction that authorities should head toward will be taking strides to build up forward-looking regulatory guidelines and lay the groundwork for regulatory harmonization. It will ultimately help dismantle barriers to entry, resulting in economies of scale and Pareto improvement and livening up the flourishing CGT market further. It is expected that international initiatives and consortiums will play a more significant role, advancing steps on an array of topics collectively. All decision-making processes should be wired to be more patient-centric and developer-friendly. Setting up communication protocols also should be prioritized so that duplicated assessments set by mutually exclusive initiatives or individual countries can be preemptively averted in advance.

Accordingly, authorities have attempted to coordinate regulatory and P&R processes, which have morphed into various forms of horizon-scanning initiatives and harmonization movements. It is intended for collectively sharing regulatory science knowledge and formulating a well-thought-out set of rules that will systematically regulate the field-not-taken. Consequently, harmonization in the realms of regulations and pricing and reimbursement is being pushed back for the sake of accessibility of innovative medicines and we can observe the converging trend where pricing and reimbursement rules of adjoining countries in the supranational union or member countries of a consortium are getting more aligned.

As for such collective movements, the European Commission is working on building the groundwork for setting up a joint European Value Framework. The Regulation (EU) 2021/2282 on HTA is a legal framework that stipulates rules on joint clinical assessments and joint scientific consultations, which replaces and formalizes EUnetHTA Joint Actions, a former joint initiative (72). The joint initiative started as an official communication framework between EMA and national/regional HTA agencies to efficiently come up with a mutually agreed upon set of rules that will override misaligned and divergent rules set by individual HTA agencies. The European Commission will further work on articulating the particulars of implementation rules under the unified goal of advancing access to innovative medicines.

The Food and Drug Administration and EMA also have embarked on a parallel scientific advice program, where authorities from both sides provide scientific guidance to developers who are considering entering both markets. The program will be especially helpful for the issues about which guidelines do not delineate in great detail or the directions or explanations from guidelines conflict with each other significantly. It provides developers official routes to actively engage with authorities on the issues that can arise from earlier stages of development.

Furthermore, consortiums have been launched to conduct joint HTA assessments and collectively rule on pricing and reimbursement. For example, FINOSE, a HTA collaboration initiative among Finland, Norway, and Sweden, conducted the joint clinical and cost-effectiveness assessment for Zynteglo^®^ (73). BeNeLuxA, a consortium among Belgium, the Netherlands, and Ireland, jointly worked on the pricing and reimbursement assessment process of Zolgensma+, with Austria participating as an external reviewer for the Belgian reimbursement assessment (74). As recently as October 2022, BeNeLuxA completed their first joint HTA report for Libmeldy^®^ and is working on laying grounds for reimbursement and pricing agreements. Pretty much in the same vein, South Korea has also announced a series of government initiatives to embark on horizon scanning initiatives with Taiwan, Canada, and Australia in the near future.

## 6. Future perspectives

Regulatory frameworks are prepared to facilitate R&D and investment environments and to act as systematic and consistent guidelines. There are still numerous regulatory issues to be agreed upon, from minuscule issues to significant ones (Table 8). Policy priorities should be placed on initiatives to harmonize with other advanced medical authorities and lay out forward-looking policies at opportune times to improve the rights of patients and the uncertainty of developers.

**Table 8:** Regulatory elements to get across and harmonize (5, 30, 31, 44, 45, 75–77).

**Area**			**Details**
Regulation	Surrogate endpoint		- FDA officially posted surrogate endpoints that were adopted as the basis of the drug assessment process on their website. - Making a list of surrogate endpoints public will benefit drug developers, which is tantamount to being transparent about assessment standards of conditional approvals
	Guideline		- Traditionally, either primarily FDA or ICH or sometimes EMA have been the ones who always set foot first on new regulatory frontiers in the biopharmaceutical sector, leading others by publishing guidelines first and setting out legislative examples. - South Korea and the EU abide by international standardizations such as ICH and PIC/S guidelines by aligning their own versions to those international standards. - South Korea and the EU purposefully aim to be active members that contribute to substances and set out standards at the forefront. - In August 2022, South Korea published the world's first kind of guideline on the assessment of mRNA-based gene therapy products. - Need to improve clarity, consistency, and transparency of assessment standards from the preclinical stage up to the post-marketing stage [e.g., post-marketing requirements and commitments (PMRs/PMCs)]. - Agreements on the robustness of clinical data that includes standards on single-arm trials and appropriate treatment sample size need to be achieved: CGT pipelines targeting rare diseases are more likely to have smaller, non-blinded, and non-randomized trials.
	Therapeutic use of investigational drug		- South Korea announced that critically-ill patients would be conditionally allowed to take foreign investigational drugs that haven't passed through the domestic approval process for ABP clinical trials. - Currently, regulations that connect clinical researches for advanced regenerative medicines with ABP clinical trials for commercial development are under study in South Korea. - Regulations on investigational drugs and hospital exemptions are not aligned across European countries yet.
	Joint value framework		- The Regulation (EU) 2021/2282 on HTA stipulates rules on joint clinical assessments and joint scientific consultations, which replaces and formalizes EUnetHTA Joint Actions, a former joint initiative. The European Commission will further work on articulating the particulars of implementation rules under the unified goal of advancing access to innovative medicines. - Joint initiatives can come up with a mutually agreed-upon set of rules that will override misaligned and divergent rules set by individual HTA agencies.
Pricing and reimbursement	Provisional reimbursed access (off-label use)		- Before official pricing and reimbursement decisions are announced, governments let firms sell at provisional terms, and price discrepancies are assumed to be settled ex-post. - France, Italy, and Germany have such routes available.
	Harmonization		- European countries have launched consortiums, FINOSE and BeNeLuxA, to collectively decide on pricing and reimbursement rules. - In July 2022, South Korea also announced its plan to launch Horizon Scanning Initiatives with Taiwan, Australia, and Canada by taking European examples after. - Sharing regulatory science knowledge and particulars of MEAs and RWD systems and outcomes of those will lead to Pareto improvements as a whole.
		RWD/RWE	- European countries have operated CAR-T RWD/RWE registries (e.g., EU's EBMT (the European Society for Blood & Marrow Transplantation), France's DESCAR-T, and Spain's VALTERMED) for the purpose of (1) post-market drug safety monitoring (2) P&R reassessment (3) researches since 2019. EMA plans to build a pan-European RWD registry. - South Korea announced an initiative to systemize the registry
		MEAs	- MEAs can be partitioned into two modes: outcome-based reimbursement and total expenditure-bound reimbursement. - Outcome-based payment approaches also have been adopted to alleviate clinical uncertainties: Spain and USA have a payment protocol available where patients pay after months-after administrations, and up to 100% refunds are backed upon unsuccessful clinical responses.

ABP, advanced biological products; CAR, Chimeric Antigen Receptor; EMA, European Medicines Agency; FDA, Food and Drug Administration; ICH, International Council for Harmonization; MEA, Managed Entry Agreements; RWD, real-world data; RWE, real-world evidence.

First, efforts to harmonize regulatory frameworks will enable participants to tap shared regulatory science. Consequential riddance of technical and regulatory barriers will align markets with pooled markets, bringing economies of scale and increased patients' access to new innovative drugs.

The issues to be aligned include practice guidelines along the clinical stages, such as good laboratory practices, good clinical practice, GMP, assessment guidelines for new modalities, platform technologies, indications, and follow-up rules that include standards on feeding real-world evidence (RWE) back into P&R assessment framework. Standards on the preclinical stage and post-marketing stage also need to be clarified, in consistent and transparent wording. Other technological and regulatory particulars include rules and standards on surrogate endpoints and therapeutic use of investigational drugs (i.e., hospital exemption). Pooled decisions can also further the P&R decision-making process by aligning terms on provisional reimbursement use, the ICER thresholds, extrapolation methods to derive long-term survival based on clinical results, discounting rates to compute benefits and costs in present values, and CEA exemption rules. Subtleties of MEAs and real-world data (RWD) systems such as their structure and constituent dataset, and rules on sharing those outcomes also need to be worked on (56). Qui et al. (78) is a comprehensive literature that presents in-depth discussions on the subsets of aforementioned elements.

Second, a forward-looking regulatory framework must be prepared in advance to achieve prior resolution of uncertainties faced by drug developers because technological realms keep branching out into new modalities. It implies that two-way communications between drug developers and authorities need to be institutionalized in such an ever-changing technical field. Patients also should be given more central roles in the design of clinical trials and P&R.

Finally, the Biden administration's recent executive order showed that an increasing number of advanced countries would shift more attention toward bio industries, following years of strenuous efforts to preserve their technological hegemony in the semiconductor and automobile industries and placing more emphasis on attaining technological hegemony in advancing biotechnologies (79). Accordingly, further regulations and industrial policies targeted at innovative drugs will be pushed for the sake of biotechnological edges and bioeconomy that follows job creations, showing a positive sign to the cell and gene therapy industry.

Well-designed industry policies that include economic incentives, such as publicly funded mega grants, tax credits for R&D and facility investment, longer market exclusivity, and research grants, will help pave the way for another stream of biotechnological innovations because many cell and gene therapy developers come from small and mid-sized biotech companies. Incentives, inversely scaled with the total number of the patient population, may also compensate for commercial uncertainties, especially in the field of rare diseases (80).

## Author contributions

SL contributed to the study design, data collection, statistical analysis, manuscript development, and review. JL supervised the entirety of the study, from study designing, data collection and analysis, and manuscript editing to manuscript submission. All authors have read and approved the final version of the manuscript.
